# Oxidative Stress: An Intersection Between Radiation and Sulfur Mustard Lung Injury

**DOI:** 10.1017/dmp.2023.238

**Published:** 2024-05-06

**Authors:** Brian J. Day

**Affiliations:** Department of Medicine, National Jewish Health, Denver, CO, USA

**Keywords:** catalytic antioxidants, mechanisms, medical countermeasures, reactive oxygen species

## Abstract

Nuclear and chemical weapons of mass destruction share both a tragic and beneficial legacy in mankind’s history and health. The horrific health effects of ionizing radiation and mustard gas exposures unleashed during disasters, wars, and conflicts have been harnessed to treat human health maladies. Both agents of destruction have been transformed into therapies to treat a wide range of cancers. The discovery of therapeutic uses of radiation and sulfur mustard was largely due to observations by clinicians treating victims of radiation and sulfur mustard gas exposures. Clinicians identified vulnerability of leukocytes to these agents and repurposed their use in the treatment of leukemias and lymphomas. Given the overlap in therapeutic modalities, it goes to reason that there may be common mechanisms to target as protective strategies against their damaging effects. This commentary will highlight oxidative stress as a common mechanism shared by both radiation and sulfur mustard gas exposures and discuss potential therapies targeting oxidative stress as medical countermeasures against the devastating lung diseases wrought by these agents.

Lethal ionizing radiation exposures lead to the development of a number of radiation sickness syndromes that occur in stages over time.^1^ The first effects seen after radiation exposure occur in the bone marrow and manifest as a leukopenia leading to increased risk of infection and bleeding.^2^ In fact, it was this observation clinicians connected the similarity of effects seen in individuals exposed to mustard gas and ionizing radiation that led to coining the term of mustard agents being “radiomimetic” and their use in treating patients with lymphomas and leukemias.^3-5^ The second syndrome involves the gastrointestinal tract leading to destruction of the gut mucosal, diarrhea, vomiting, and nutrient malabsorption.^6,7^ A third syndrome affects the lungs, resulting in lung edema and fibrosis.^8-10^ Mustard gas exposures also lead to many of the same syndromes reported with radiation exposures. Mustard gas exposures result in leukocyte depletion and the development of lung edema and fibrosis.^11,12^ Early studies on ionizing radiation chemistry played a fundamental role in the creation of the field that studies free radicals in biological processes and the body’s defense against their potential deleterious effects on cellular macromolecules.

## Free Radical Biology and Oxidative Stress

**Figure 1.**
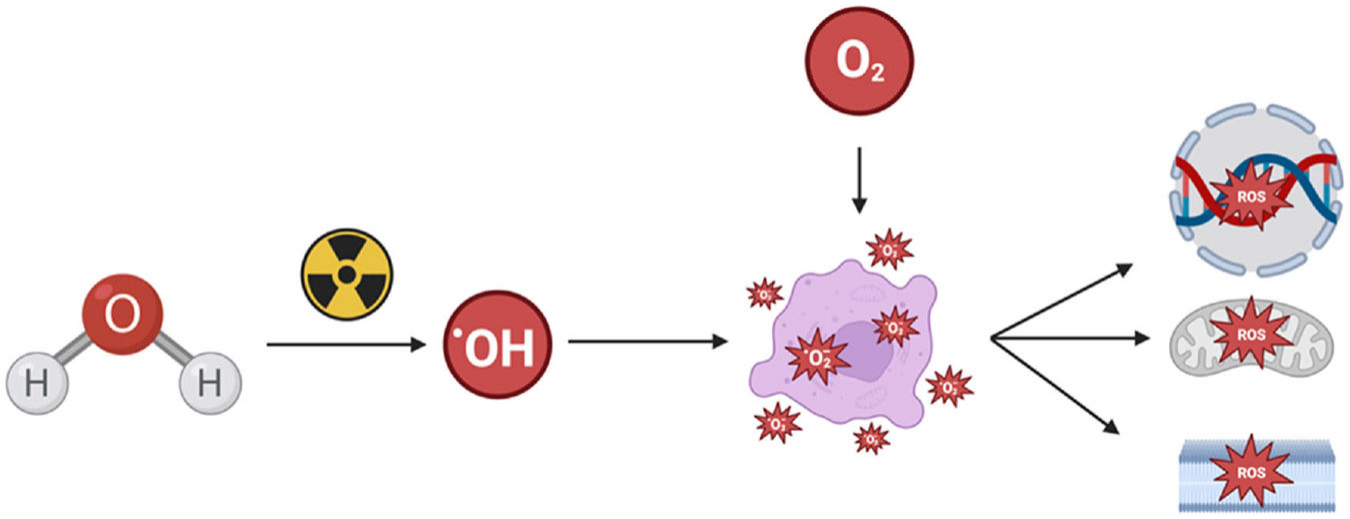
Ionizing radiation reaction with water generates hydroxyl radical and, in the presence of oxygen, enhances cell injury through reactive oxygen species (ROS) that damages DNA, lipid membranes, and mitochondria.

Ionizing radiation is a well-known source of oxidative stress that leads to a series of deleterious events. Ionizing radiation splits water (H_2_O) generating the very reactive hydroxyl radical (·OH).^13^ Given that tissue is mostly water, this is a major source of reactive oxygen species (ROS) associated with ionizing radiation exposures (Figure 1). A free radical, such as the hydroxyl radical, rapidly reacts with non-radical molecules that generate a new free radical in a chain reaction that is propagated by oxygen.^14,15^ This type of rapid reaction with cellular macromolecules limits diffusion, whereas other ROS with slower reaction kinetics can often diffuse and damage a target distance from the initial reaction. Extensive work in this area helped develop the understanding that ROS can play either a beneficial or harmful effect in biological processes. Factors that dictate whether ROS production produces a beneficial or harmful effect include location, type of ROS produced, source, location, target affected, concentration produced, duration of production, and surrounding antioxidant defenses.

The body is continuously exposed to sources of oxidants generated endogenously and exogenously. An older concept of oxidative stress was the imbalance of oxidant generation and defense where defenses are overwhelmed, and oxidation of cellular macromolecules ensues. However, it is increasingly known that normal cellular functions require oxidation events and there are redox switches involved with normal cellular homeostasis.^16^ Some examples of redox switches are found in the cell signaling pathways of Nrf2,^17^ NF-kB,^18^ and the HIF^19^ systems. Oxidative stress is better defined as oxidation events that over oxidizes these redox switches, preventing their ability to be reduced back and thus disrupting normal homeostasis.^20^ Several ROS have been shown to participate in these types of reactions. Superoxide is a ROS that is formed by the addition of an electron to oxygen. Endogenous generation of superoxide occurs during the uncoupling of oxidative phosphorylation in the mitochondria and is increased by hyperoxia.^21^ There are also several enzymatic systems that can directly generate superoxide such as the NADPH oxidases (NOXs)^22^ and many cellular oxidoreductases that generate superoxide as uncoupling events.^23^ Superoxide is rapidly dismutated spontaneously to hydrogen peroxide or enzymatically by a family of superoxide dismutases (SODs) to hydrogen peroxide.^24-26^ An important caveat of the reactivity of superoxide is that, wherever superoxide is formed, there will also be hydrogen peroxide. Hydrogen peroxide readily reacts with cellular thiols resulting in the formation of a series of thiol oxidation states.^27^ Many of these thiol oxidation states can be reduced by cellular reductants, but the higher the oxidation state, the less readily they can be reduced, leading to a state of oxidative stress and disruption of cellular homeostasis.^28^

**Figure 2.**
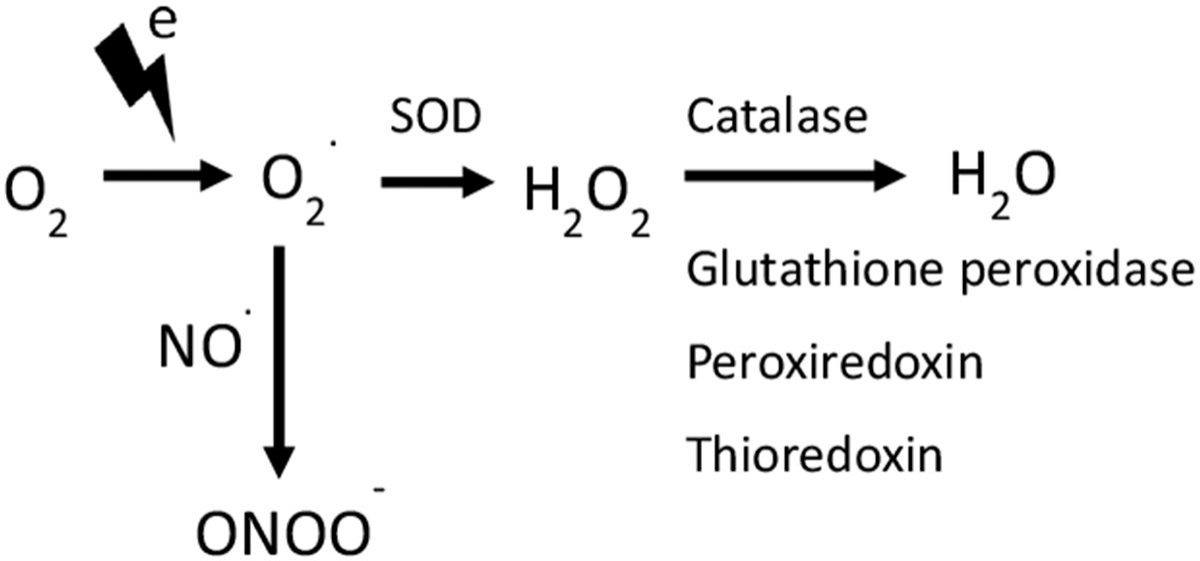
Addition of electrons to oxygen generates a series of reactive oxygen species, including superoxide and hydrogen peroxide. Superoxide reaction with nitric oxide generates peroxynitrite. Superoxide dismutases (SOD) and catalase work together to convert reactive oxygen species to water. The body has several other antioxidant enzyme systems that convert hydrogen peroxide to water, including glutathione peroxidases, peroxiredoxins, and thioredoxin.

The discovery of cellular antioxidant defenses also contributed to our understanding that the body can defend against the adverse effects of ROS produced by exogenously and endogenously derived sources.^29^ Some of these endogenous antioxidant enzymes work in parallel to metabolize superoxide to hydrogen peroxide and hydrogen peroxide to water (Figure 2). These endogenous enzymes include the SODs, catalase, and glutathione peroxidases. In addition, several small proteins also contribute to the metabolism of hydrogen peroxide to water, including glutathione, peroxiredoxins, thioredoxin, and the enzyme systems that recycle them such as glutathione reductase and thioredoxin reductase. Small molecules, such as manganese porphyrins that mimic the effects of endogenous antioxidant enzymes, are of benefit in treating conditions involving oxidative stress, including radiation and sulfur mustard-induced lung injury.^30^

The role of oxidative stress underlining lung injury responses and disease has been extensively characterized.^31^ Prolonged lung exposure to high oxygen concentrations leads to oxidation of cellular macromolecules and pulmonary edema.^32^ Hyperoxia-induced lung injury can be attenuated by the overexpression of SOD implicating the role superoxide plays in producing lung injury.^33^ Several pulmonary toxicants, such as paraquat and bleomycin, can lead to the elevation of superoxide and hydrogen peroxide production and oxidation of cellular macromolecules associated with lethal pulmonary edema and fibrosis.^34-36^ Studies have shown resistance against lung edema and fibrosis from these types of agents correlates with overexpression of endogenous antioxidant enzymes.^37-40^ Conversely, animals are more sensitive to deleterious effects of these types of agents when antioxidant enzymes are depleted or knocked out.^41,42^ Catalytic antioxidants are also effective in attenuating damage from paraquat and bleomycin-induced lung injury.^43,44^ These studies suggest that agents that elevate levels of ROS species can produce lung injury and may be a potential mechanistic target to treat lung injury.

## Oxidative Stress in Radiation-Induced Lung Pathology

**Figure 3.**
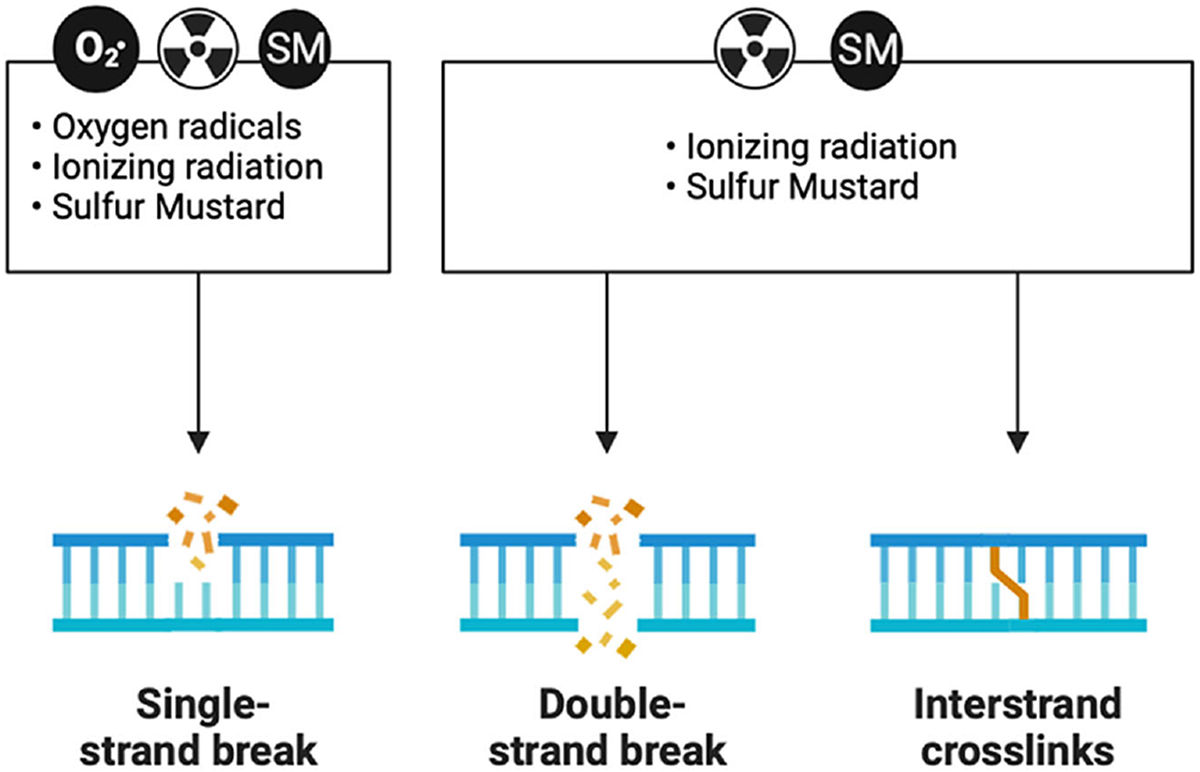
DNA damage is a shared mechanism between reactive oxygen species, ionizing radiation, and sulfur mustard (SM). All three moieties can produce DNA single-strand breaks. Both ionizing radiation and SM can produce DNA double-strand breaks and DNA crosslinks.

Experiments exposing cells to ionizing radiation in the presence and absence of oxygen gave rise to the idea that this process was important in driving the deleterious effects of ionizing radiation.^45,46^ The observations that oxygen potentiated the damaging effects of radiation suggested that secondary ROS also plays a role in the damaging effects of radiation.^47^ The disconnect between the rapid chemistry of ionizing radiation with cellular macromolecules and the delayed onset of cellular dysfunction suggests the presence of secondary longer lived ROS.^48^ Ionizing radiation exposures are associated with damage to DNA resulting in DNA strand breaks and resultant mutagenic events.^49,50^ DNA damage is a shared common mechanism of injury response with sulfur and nitrogen mustards (Figure 3). Some of these events are driven by oxidation of nucleic acids that produce base-pair mismatches.^51,52^ Ionizing radiation exposures in the presence of oxygen stimulate tissue injury, whereas tissues with low oxygen tension are more resistant to radiation-induced injury.^53-56^ Studies have found cell resistance against ionizing radiation-induced cell death correlates with the expression levels of endogenous antioxidant enzymes.^57^ SOD is one of the antioxidant enzymes found early on to be protective against radiation-induced damage.^58^ Depletion of these antioxidant systems has been shown to sensitize animals to the effects of ionizing radiation and, conversely, their overexpression can protect animals from the deleterious effects of ionizing radiation.^59^ One of the first antioxidants to be approved by the FDA was amifostine for the treatment of tissue injury due to radiation treatments.^60^ This compound is typically used prophylactically to reduce late effects produced by radiation therapy. Amifostine has been shown to protect against the formation of pneumonitis and fibrosis in the mouse lung.^61^ However, its need to be used before radiation exposure and its narrow window of efficacy limit application as a medical countermeasure for ionizing radiation.

**Figure 4.**
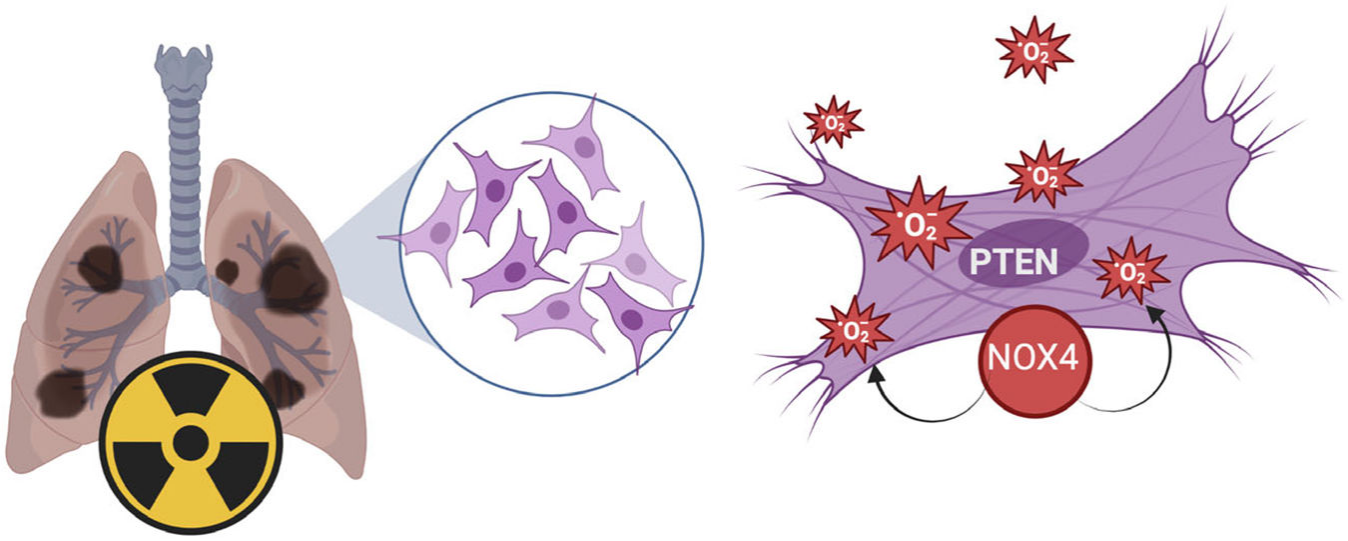
Potential feedforward mechanism involving a role of reactive oxygen species in radiation-induced lung fibrosis. Apoptotic resistant myofibroblasts are involved in radiation-induced lung fibrosis that is orchestrated in part through a feedforward loop of upregulation of phosphatase and tensin homolog (PTEN) and NADPH oxidase 4 (NOX4) expression resulting in the production of superoxide.

Ionizing radiation can produce lung pleural effusions and pneumonitis that can progress to lung fibrosis. Some of these effects can be species- and strain-specific.^62^ Some mouse strains present with different acute responses to ionizing radiation.^63^ Several biological pathways have been found to be disrupted by ionizing radiation. These include inflammatory pathways associated with changes in cytokines and growth factors, fibrotic pathways, tissue injury and repair pathways, and oxidative stress pathways. Recent findings suggest that hypoxia-regulated genes are associated with early changes in oxidative stress markers in irradiated lungs of mice.^64^ These pathological changes are associated with the accumulation of markers of oxidative DNA damage.^64-66^ Fibroblasts in fibrotic foci have elevated levels of NOXs that produce superoxide. These myofibroblasts are resistant to apoptosis and are thought to drive remodeling of the interstitial tissue producing fibrotic tissue.^67,68^ Studies have reported that NOX4 is upregulated in the lung from 1 day to 6 months after radiation exposure and may be linked to phosphatase and tensin homolog (PTEN) expression driven by its promoter being hypomethylated due to reactive oxygen species generation in a feed-forward mechanism^66^ (Figure 4).

## Oxidative Stress in Sulfur Mustard-Induced Lung Pathology

Sulfur mustard gas exposures have been shown to injure the eyes, skin, and lung.^69^ The degree and severity of organ injury depend upon the route of exposure in animal models. Lung oxidative stress in sulfur mustard-induced injury has been shown after subcutaneous injection.^70^ More commonly, lung models of sulfur mustard gas exposures use a lung intubation model of mustard gas delivery that produces a robust bronchial lung vasculature leak and subsequent fibrin cast formation.^71,72^ One of the first examples for a role of oxidative stress in sulfur mustard-induced lung injury involved studies where lung instilled with antioxidants attenuated lung injury in rats exposed to the half mustard 2-chloroethyl sulfide (CEES).^73^ Biopsies from humans exposed to sulfur mustard show increased expression of enzymes known to elevate the levels of endogenous ROS and correlate with increased markers of oxidative stress in bronchoalveolar lavage fluid.^74^ The alkylating and cross-linking effect of sulfur and nitrogen mustards on cellular macromolecules are well studied and thought to be the initiating mechanisms in cell injury responses like those described in ionizing radiation (Figure 3). The mechanisms by which sulfur and nitrogen mustards generate ROS and potential sources of generation have not been as widely studied as they have been in radiation.

A few reports in the literature suggest that cultured cells exposed to sulfur mustard result in mitochondrial damage leading to mitochondrial generation of ROS.^75,76^ Sulfur mustard may trigger release of damage-associated molecular patterns (DAMPS) from injured cells that may signal through TOLL-like receptors (TLRs) activating NOXs and other inflammatory mediators. Activation of NOXs in fibroblasts could be involved in fibrotic mechanisms resulting in sulfur mustard-induced lung fibrosis as has been described in radiation (Figure 4). Tissue resident macrophages and circulating macrophages and neutrophils also contain NOXs that can be activated by sulfur mustard-induced tissue damage. Another reactive species that may play a role in sulfur mustard injury responses are reactive nitrogen species through the activation of nitric oxide synthetase (NOS) and formation of nitric oxide (NO·). Pharmacological inhibition of NOS has been shown to attenuate nasal injury in rats exposed to the half mustard CEES.^77^ NO· can rapidly react with superoxide to form the more reactive peroxynitrite that can modify cellular macromolecules, including proteins, lipids, and nucleic acids.^78^ Mice deficient in NOS have also been reported to be less sensitive to CEES-induced injury and inflammation.^79^ There are numerous potential mechanisms for sulfur mustard to generate ROS, and many of these are shared with ionizing radiation exposures.

## Development of Catalytic Antioxidants

The term *antioxidant* is a broad term given to endogenous and exogenous agents that can interact with reactive species at sufficient rate constants that result in less reactive product(s). Many compounds meet this requirement, but when given to cell systems or whole animals they have little effects or require large amounts to produce mild affects. The reason for this is the low-rate constant of the reaction between the reactive species with the antioxidant and that most common antioxidants are consumed in the reaction. Both these effects limit most antioxidants, including commonly used compounds such as N-acetyl cysteine, ascorbate, vitamin E, and amifostine, which require large doses and have modest efficacy responses. These antioxidants need to be present in the system at levels that outcompete cellular macromolecules due to their inherent low rates of reaction toward the reactive species compared with cellular enzymatic antioxidant systems. Another issue with these types of antioxidants is they disrupt cellular homeostasis that relies on oxidative processes by saturating the cell with reducing agents.^23,80^

Some of the cellular enzymatic antioxidant systems use transition metals in their active sites that can readily accept and give electrons to reactive species leading to dismutation reactions that recycle the enzyme back to its resting state and are not consumed in the reaction. SODs contain iron, copper, or manganese in their active sites and cycle between reduced and oxidized states.^81^ Superoxide gives an electron to SOD and is converted back to oxygen while another superoxide molecule accepts an electron from the SOD and is converted to hydrogen peroxide. A similar process occurs with catalase where hydrogen peroxide gives 2 electrons to catalase forming oxygen and catalase gives 2 electrons to another hydrogen peroxide molecule forming water. In both cases, the transitional metals return to their resting state and are ready for another round of reactions.

**Figure 5.**
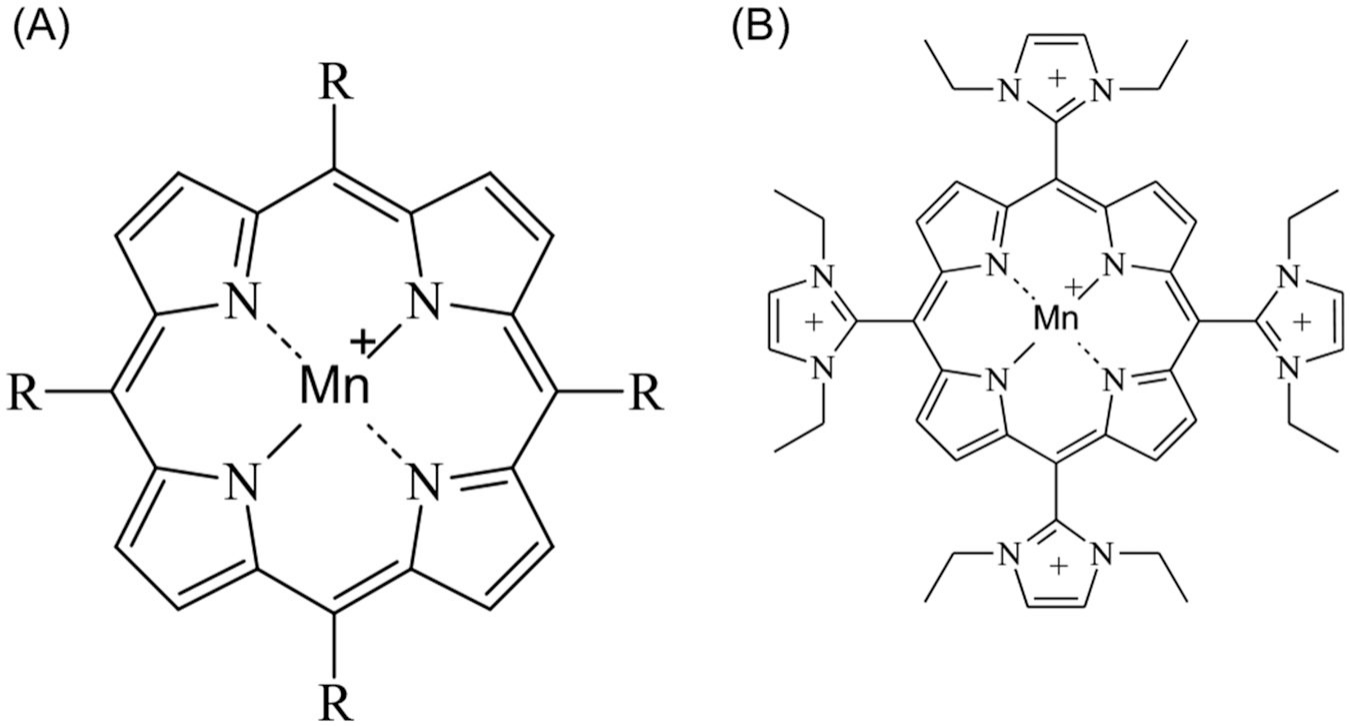
(A) Pharmacophore for manganese meso-porphyrins that are broad spectrum catalytic antioxidants. (B) Chemical structure for AEOL10150 (manganese [III] meso-tetrakis [N, N’-diethylimidazolium-2-yl] porphyrin).

There have been several metal-containing complexes developed as SOD and catalase mimetics.^30^ Most of these compounds complex the metal in a ring-like structure that alters the redox potential of the metal. The redox potential drives the rate of reaction with the reactive species. The fastest rate of reaction occurs when the redox potential sits halfway between the 2 half reactions, where neither half reaction is rate limiting. This is generally true for endogenous antioxidant enzymes like CuZnSOD. Other important properties of these antioxidant metal complexes are the affinities of the ring structures for the metal, solubility of the complex, and toxicity. One class of these types of antioxidants is the manganese *meso*-porphyrins (Figure 5A). This class of antioxidants has been shown to protect cultured cells and animals against several ROS or compounds that generate ROS.^82^ The *meso*-porphyrins are a useful platform that can be modified by changing the 4 pendant groups or using different transition metal chelates. For example, some metals complexed in the same ligand can have dramatically different toxicities. For example, the metal ligand tetrakis (4-benzoic acid) *meso*-porphyrin (TBAP) when complexed with manganese (MnTBAP) has a LD_50_ in mice of 101 mg/kg, ip, with a 95% confidence interval of 104–98 mg/kg and when iron is complexed in this ligand (FeTBAP) has an LD_50_ of 31 mg/kg, ip, with a 95% confidence interval of 45–22 mg/kg (unpublished data). The metal ligand can also influence the potency of the compound. A comparison of 4 different metals in a lipid peroxidation assay found IC_50_s ranged from 20–950 μM for the TBAP ligand with Co = Mn>>Fe>>Zn.^83^ The metalloid *meso*-porphyrins were also found to be 100 times more potent in the lipid peroxidation assay than the vitamin E analog trolox and flavonoid rutin and 10 times more potent than CuZnSOD.^83^ Many of these metal complexes have shown efficacy in several animal models of human disease.^30^ This class of antioxidants have also been shown to be effective in animal models of radiation-induced injury,^84-88^ and a few have been efficacious in sulfur mustard-induced injury.^77,89-91^

## Therapeutic Effects of Catalytic Antioxidants in Radiation and Sulfur Mustard-Induced Lung Pathology

Amifostine is one of the few antioxidants approved for human use as a treatment of radiation-mediated injury. So, the concept that antioxidants could be beneficial against radiation-induced injury is not new. In fact, there are numerous reports in the literature of positive effects of antioxidants in animal models of radiation-induced lung injury.^92-98^ Likewise, there are several publications showing the positive effects of a wide range of antioxidants in animal models of sulfur mustard-induced injury.^77,89-91,99-106^ The literature supports a scientific premise of antioxidants having dual efficacy in both radiation and sulfur mustard-induced lung injury.

One group of antioxidants that have shown dual efficacy in animal models of both radiation and sulfur mustard-induced lung injury is the manganese *meso-*porphyrins. The manganese *meso-*porphyrin AEOL10150 (manganese [III] tetrakis N,N’-diethylimidazolium-2-yl porphyrin) (see Figure 5B) has been extensively characterized as a potent broad spectrum antioxidant.^107^ This compound has been deployed in several rodent and primate models of radiation-induced lung injury.^85-87,108-111^ These studies reported benefits of AEOL10150 against radiation-induced pleural effusions, injury, and fibrosis in animal radiation models. In addition, markers of oxidative damage were also attenuated in the lungs of animals treated with AEOL10150. In one of these studies, mice were exposed to 15Gy whole thorax radiation, and apoptotic signaling was determined 6 weeks after radiation.^66^ This study reported apoptosis in lung type I and type II cells and endothelium that correlated with PTEN expression, increased tumor suppressor protein 53 (p53), Bcl-2-associated X protein (Bax), transforming growth factor-beta (TGF-β), and NOX4 expression along with markers of oxidative stress that were all attenuated in mice receiving AEOL10150 treatment. Studies involving lethal whole thorax radiation in rhesus macaques found similar increases in lung tissue levels of p53, PTEN, Bax along with adhesion molecules and markers of oxidative stress and those receiving AEOL10150 had attenuated levels of some of these markers.^108^ Another study of rhesus macaques receiving whole thorax lung irradiation and a subset given AEOL10150 24 hours post-radiation had improved survival and diminished quantitative radiographic lung injury as determined by CT-scans.^111^ Another report in non-human primates exposed to radiation and given AEO10150 had evidence of less need for steroid support, improved radiographic evidence of lung damage, and survival.^109^

Early screening of manganese *meso*-porphyrins against CEES-mediated injury in cultured human epithelial cells identified AEOL10150 as a promising sulfur mustard mitigating agent.^75^ These studies also revealed mitochondrial oxidative stress induced by CEES exposure and the ability of AEOL10150 to attenuate CEES-mediated mitochondrial oxidative stress. AEOL10150 has been shown to attenuate nasal and lung injury in rat models of sulfur mustard exposure.^77,89,90^ Early studies using a nose only exposure to 5% CEES produced substantial injury to both the nasal cavity and the lungs of rats. AEOL10150 given by intranasal and subcutaneous injection rescued nasal epithelial injury and markers of oxidative stress in the nose only CEES inhalation model.^77^ Another study using a nose only exposure to 5% CEES showed AEOL10150’s ability to rescue lung injury when given 1 and 9 hours post CEES exposure.^90^ AEOL10150 decreased CEES-mediated increases in bronchoalveolar lavage markers of lung injury and edema along with tissue markers of oxidative DNA and lipid damage. In a lung intubated model of lethal sulfur mustard exposure in rats, AEOL10150 given by subcutaneous injections of AEOL10150 1 hour post-exposure and every 4 hours later decreased sulfur mustard-induced airway cast formation, improved blood oxygenation, clinical scores, and dramatically improved survival.^89^ These studies also reported attenuation of markers of oxidative stress and inflammation in AEOL10150 treated rats exposed to a lethal concentration of sulfur mustard gas. It is interesting to note that AEOL10150 has been shown to be able to mitigate the lung injury in several other chemical threat agents, including chlorine gas,^112^ phosgene (unpublished data), organophosphates,^113^ and nerve agents.^114^

## Conclusion

**Figure 6.**
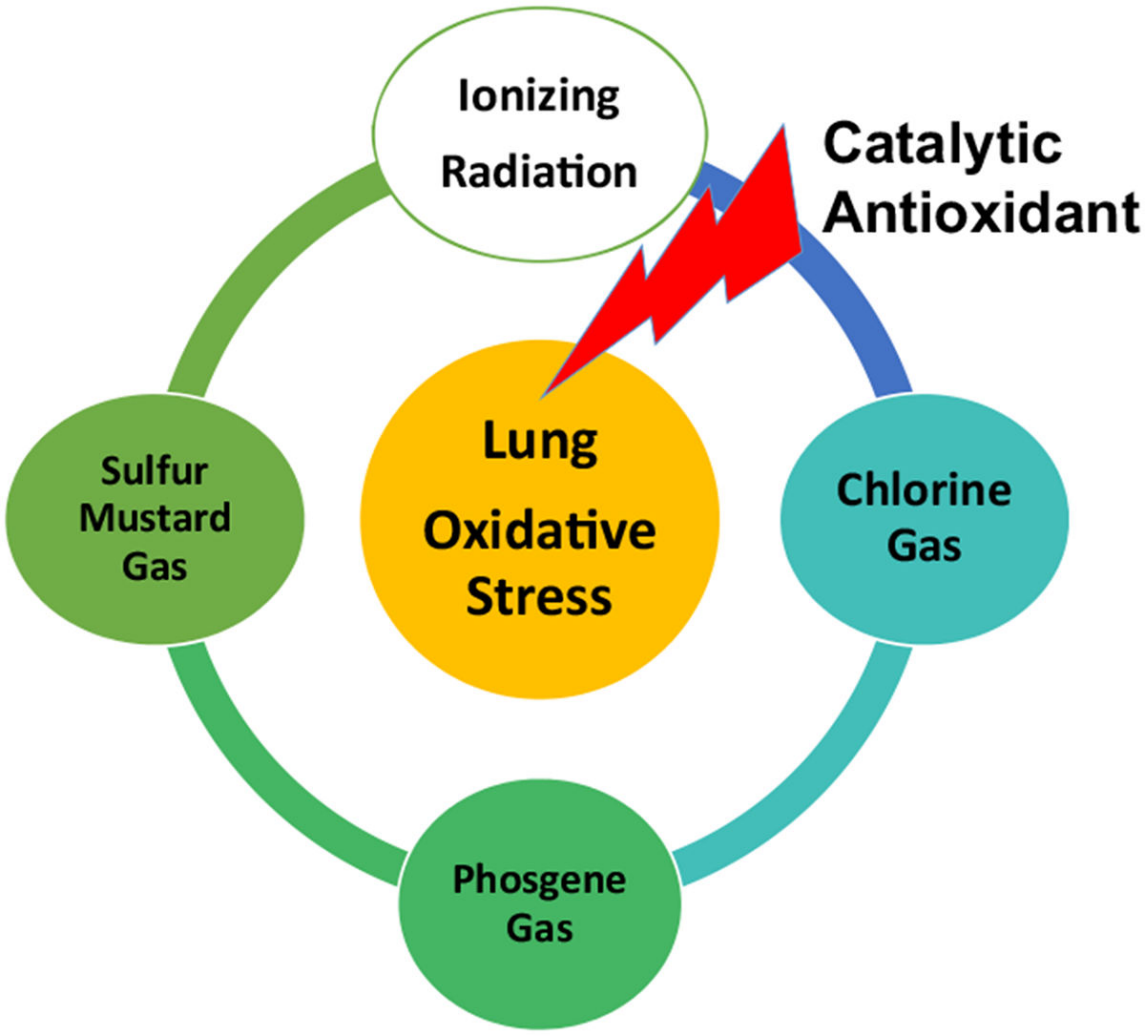
Lung oxidative stress as a common mechanistic target shared by several threat agents and catalytic antioxidants as broad spectrum medical countermeasure.

Given the large number of threat agents and the time and expense to develop individual medical countermeasures, it is prudent to identify and focus on therapeutic targets that are shared by multiple threat agents. Oxidative stress is a shared therapeutic target for the treatment of radiation and sulfur mustard-induced lung pathology. Antioxidants may provide a therapeutic class of agents that could be deployed against multiple threat agents as medical countermeasures (Figure 6).

## Abbreviations:

AEOL10150: Manganese (III) tetrakis N,N’-diethyl-imidazolium-2-yl porphyrin
Bax: Bcl-2-associated X protein
CEES: 2-chloroethyl sulfide
DAMPS: Damage-associated molecular patterns
FDA: Federal Drug Administration
HIF: Hypoxia-inducible factor
MnTBAP: Manganese (III) tetrakis (4-benzoic acid) *meso-*porphyrin
NF-kB: Nuclear factor kappa B
NOS: Nitric oxide synthetase
NOX: NADPH oxidase
Nrf2: Nuclear factor erythroid 2-related factor 2
p53: Tumor suppressor protein 53
PTEN: Phosphatase and tensin homolog
ROS: Reactive oxygen species
SM: Sulfur mustard
SOD: Superoxide dismutase
TGF-β: Transforming growth factor beta
TLR: TOLL-like receptor
